# Identifying the “Active Ingredients” of a School-Based, Workplace Safety and Health Training Intervention

**DOI:** 10.1007/s11121-021-01209-8

**Published:** 2021-01-22

**Authors:** Mikko Nykänen, Rebecca J. Guerin, Jukka Vuori

**Affiliations:** 1 Finnish Institute of Occupational Health, PO Box 40, 00032 Helsinki, Finland; 2 Division of Science Integration, National Institute for Occupational Safety and Health, US Centers for Disease Control and Prevention, Cincinnati, OH, USA

**Keywords:** Young worker, Occupational safety and health, Fidelity of implementation, Vocational education, Injury prevention

## Abstract

Young workers in many industrialized countries experience a higher rate of largely preventable occupation-related injuries compared with adults. Safety education and training are considered critical to the prevention of these incidents. This can be promoted by the dissemination and scale-out of an evidence-based, safety training programs in vocational education. The aim of this study was to identify the intervention core components that comprise the “active ingredients” of a safety training intervention for young workers and assess the impact on student outcomes of interest. Fidelity of implementation was operationalized using measures of adherence and quality of intervention delivery. For this study, data were collected through a school-based, cluster randomized trial conducted in 2015 in eight Finnish upper secondary-level vocational schools (*n* = 229 students in 22 groups, each with one teacher). Results indicate that the intervention core components (safety skills training, safety inoculation training, a positive atmosphere for safety learning, and active learning techniques) had differing associations with student outcomes. Adherence related to the acquisition of safety skills training was the strongest active ingredient in terms of positive effects. Furthermore, quality of delivery in terms of fostering positive learning atmosphere and utilizing active learning methods was associated especially with motivational outcomes. These findings indicate that different active ingredients complemented each other. Contrary to expectations, we found no statistically significant relationship between any of the core components and risk-taking attitudes. The current study advances prevention science by identifying the active ingredients of an evidence-based intervention, implemented in Finnish vocational school settings, that helps protect young workers from work-related morbidity and mortality.

## Introduction

Previous research indicates that younger workers (defined as adolescents and young adults aged under 30 years) are at an elevated risk of being injured (and in some cases fatally) at work (Breslin & Smith, 2006; Hanvold et al., 2019; Guerin et al., 2020). Inexperience, exposure to job hazards and unsafe tasks (such as heavy lifting), psychosocial factors (such as low job control), and organizational factors (such as poor safety climate) are just some of the multiple factors associated with increased injury risk for young workers (Breslin & Smith, 2006; Hanvold et al., 2019). These incidents have been shown to have long-term, negative impacts on adolescent/young adult health and development (Koehoorn et al., 2008). Vocational education plays an important role in preparing young people with knowledge and skills for safe and healthy work (Boini et al., 2017; Rodrigues et al., 2018). Guerin et al. (2019) highlighted that psychological theories provide guidance for developing school-based interventions to prepare for young people for hazards they may face in the workplace. However, it is noteworthy that there have been relatively few school-based OSH intervention studies based on psychological theories. According to a previous randomized-controlled trial (Nykänen et al., 2018, 2019), a safety training approach based on social-cognitive framework (Bandura, 1997; Rotter, 1982) and expectancy-value theory (Eccles & Wigfield, 2002) had positive outcomes among students in vocational education. However, there is need for a more thorough understanding of the processes underlying intervention efficacy and effectiveness and to identify which aspects, or components, of the intervention are the main contributors to the outcomes of interest.

Previous research has emphasized the importance of core components, derived from theory or empirical evidence, that are most likely to account for the main effects of successful interventions (Backer, 2001; Fixsen et al., 2009; Irwin & Supplee, 2012). Not all core components are created equal, with some linked to stronger intervention effects more than others (Abry et al. 2015). When the most important core components of an intervention are identified, efforts to replicate or adapt that program will be more successful because these key elements can be kept intact (Espada et al., 2012). How to identify what is essential is an important challenge in the successful implementation of evidence-based programs (Durlak & DuPre, 2008) and can be facilitated by the use of theoretical frameworks during intervention development (Allen et al., 2018). Given that the core components represent the program theory/internal logic, fidelity entails that these essential elements are implemented in the manner intended by program developers (Allen et al., 2018; Dusenbury et al., 2003; Mowbray et al., 2003). Said differently, intervention core components are the program elements hypothesized to transmit effects and therefore are the target of fidelity assessments (Abry et al., 2015).

The increased attention in prevention research on the measurement of fidelity is evidenced by the proliferation of fidelity models and measurement approaches (e.g., Berkel et al., 2011; Carroll, 2020; Dane & Schneider, 1998; Gearing et al., 2011; Schoenwald et al., 2011). Although none are definitive (Berkel et al., 2011), two common features of most fidelity frameworks and models advanced by researchers include (1) how much and (2) how well a practice was used as intended (i.e., dose or amount of program delivered, adherence to the program protocol, quality of program delivery, and participant acceptance (Dunst et al., 2013; Rohrbach et al., 2006). Efforts to test the hypothesized, essential elements of interventions are rare (Durlak & DuPre, 2008; Abry et al., 2015). Research is needed on how to identify, through empirical investigation, the so-called “active ingredients” of interventions and explore which are most responsible for improving program outcomes (Abry et al., 2015; Collins et al., 2005; Durlak & Dupre, 2008). More research in this area has also been called for in the occupational safety and health (OSH) field (Dugan & Punnett, 2017; Schulte et al., 2017). In sum, knowledge of a program’s active ingredients can be used to identify specific practices, when implemented with fidelity, that facilitate desired change in participants, to optimize intervention effectiveness, and to reduce costs and burden on program participants and implementers (Abry et al., 2015).

To address a gap in the prevention science, public health, and OSH research, we explored the associations between the hypothesized core components, implemented with fidelity, and intervention outcomes of a school-based, OSH training program, *Attitude to Work*, developed in 2015 by the Finnish Institute of Occupational Health (FIOH), to identify active intervention ingredients.

### Attitude to Work Program

*Attitude to Work* is a safety training program targeted at students in upper secondary level vocational education. The intervention was developed in collaboration with vocational schools and workplaces. The program consists of two full, consecutive training days (12 h) and is implemented by teachers. The program is publicly available online and includes a practical, easy-to-use facilitator’s guide with ready-to-use lesson plans and concrete examples of safety training activities. Train-the-trainer workshops conducted by FIOH provide pedagogical tools, resources, and professional development support for teachers. The program is highly structured, providing detailed instructional guidelines for the teacher to implement the program with a student group. The teachers also receive a two-day training session provided by FIOH before implementing the program.

The intervention mechanism is based on social cognitive theory (Bandura, 1997; Rotter, 1982) and expectancy-value theory (Eccles & Wigfield, 2002). These two theories share many similarities by addressing the role of competence-related beliefs in human behavior, but they also complement one another (see Leaper, 2011). Competence-related beliefs such as self-efficacy have received more emphasis in social cognitive theory, whereas the expectancy-value theory outlines competence related beliefs, perceived cost, and utility as important behavioral determinants. Perceived cost refers to negative aspects of engaging in the behavior, and perceived utility refers to the usefulness of the activity (Leaper, 2011; Bandura, 1997; Eccles & Wigfield, 2002). Hence, a combination of these theories may offer a more comprehensive framework for influencing antecedents of human safety behavior and provide guidance for developing educational safety interventions. In line with the key aspects of social-cognitive theory and expectancy theory, the *Attitude to Work* safety training program stresses personal control over safety, focuses on competence-related beliefs, and guides students to identify the positive outcomes of preventive actions and negative consequences of risk-taking. Moreover, *Attitude to Work* promotes a pedagogical approach to safety learning that includes active participation on the part of learners (e.g., modeling, feedback, reflection and dialogue) (see Burke et al., 2006).

In the current study, we assessed the *Attitude to Work* program in terms of four core components that are hypothesized to represent the underlying intervention mechanism. Fidelity thus requires that these core elements are implemented, as designed, during program delivery. Major features of the intervention are based on its (a) educational content that focuses on safety skills training, (b) educational content that focuses on safety inoculation training, (c) utilizing active learning methods, and (d) fostering positive atmosphere for safety learning. These core components represent the internal logic of the intervention and can be measured to identify intervention program active ingredients serving as key levers of change (Abry et al., 2015). Next, we provide a description of the core components of *Attitude to Work* training program.

### Safety Skills Training

Safety skills training involves identifying hazards at the workplace, analyzing the factors that precede incidents and the relationship between unsafe behavior and work-related morbidity and mortality, identifying behavioral strategies for preventing injuries and illnesses, learning about the negative consequences of staying silent about safety issues and the positive consequences of information-seeking and speaking about safety at work, and setting personal, occupational safety goals. During the safety skills training, students are guided toward identifying controllable causes of work-related morbidity and mortality and recognizing personal control over safety.

### Safety Inoculation Training

This core component is related to practicing how to act when encountering coworkers’ risky behavior at the workplace, unfamiliar work tasks, or unsafe work situations. The key idea in safety inoculation training is that the students are guided to identify behavioral strategies to overcome barriers to safe work. Students reflect on possible solutions to challenges and how to implement them in practice. Students are guided to acknowledge their own personal opportunities and means of overcoming challenges that undermine safe work.

### Fostering a Positive Atmosphere for Safety Learning

The third intervention component promotes safety learning through emotional motivation and fostering active and engaging learning environments. This intervention component supports peer reinforcement during training activities and facilitates emotional motivation in safety learning. Intervention guidelines provide teacher instructions on how to foster a supportive, respectful, and engaging learning environment during program implementation.

### Use of Active Learning Techniques

Instructional techniques are based on the learner’s own active participation. The program promotes interaction and dialogue between students as they share their safety-related experiences, knowledge, and skills. The intervention program includes group discussions, role play and small-group tasks, and problem-solving exercises. Instead of lecturing, the trainers use the knowledge, ideas, and experiences of the participants themselves as part of the learning process.

### Earlier Findings on Intervention Impact

Results from previous research (Nykänen et al., 2018, 2019) indicate that *Attitude to Work* had beneficial outcomes in terms of safety preparedness, the internal safety locus of control, risk attitudes, and the safety motivation of students in vocational education. While previous studies demonstrated positive intervention effects, to enhance research-to-practice in real-world settings, it is important to gain an understanding of the key elements or mechanisms that transmit these outcomes. Next, we provide a brief overview of the personal safety competencies to which the intervention program is targeted.

Nykänen et al., (2018) defined safety preparedness as “young peoples’ readiness to implement actions that support occupational safety, and their resilience to deal with barriers or problems related to occupational safety and safe working” (p. 46). Safety preparedness is a cognitive construct comprising safety self-efficacy and preparation for barriers for safe work. Safety-related self-efficacy refers to the degree of confidence in one’s ability to perform safety-related activities successfully at workplace, such as acquiring instructions or guidelines at work in order to work safely. Preparation for barriers for safe work refers to abilities to anticipate potential barriers and utilize behavioral strategies to resolve the respective problematic situations (Nykänen et al., 2018). The internal safety locus of control refers to perceived control over occupational morbidity and mortality (Jones & Wuebker, 1985). Both Internal safety locus of control and safety-related self-efficacy focus on competence-related beliefs, but each from a different perspective. Safety locus of control refers to a general perception of personal control over work-related incidents. Self-efficacy in turn refers to one’s perceived ability to effectively perform specific behavioral activities (see Nykänen et al., 2019). Safety motivation has been defined as “an individual’s willingness to exert effort to enact safety behaviors and the valence associated with those behaviors” (Neal & Griffin, 2006. p. 947). Risk attitudes have been defined as the extent to which participants view occupational safety-related risk-taking at the workplace as appropriate (see Nykänen et al., 2018).

### Current Study

This study expands previous research by exploring associations between the core components of the *Attitude to Work* intervention and the targeted outcomes. Safety skills training and safety inoculation training utilize two learning mechanisms: mastery experiences of safety promotion activities and vicarious learning through social modeling of other students’ performance during safety training activities. Bandura (1997) has highlighted that these two learning mechanisms are important sources of self-efficacy. Also, safety skills training guides students to identify the negative outcomes (e.g., work-related injuries and illness) of unsafe behavior at work and positive outcomes (e.g., avoiding injuries and illness) of preventive actions. Similarly, safety inoculation training stresses the importance of employee’s own positive attitudes and actions in terms of overcoming barriers for safe work. According to expectancy value (Eccles & Wigfield, 2002), the relative value that individuals place on certain activities and the perceived cost of certain actions are important determinants of attitudinal change. Thus, delivering safety skills training and safety inoculation training with high fidelity may contribute to positive, attitudinal outcomes of the program. Safety skills training and safety inoculation training activities also focus on perceptions of personal control over work-related injuries and illness. Reorienting control beliefs may have an impact on internal safety locus of control (see Huang & Ford, 2011). Previous research (Nykänen et al., 2019) indicates that modifying competence-related beliefs contributed to the intervention effect on safety motivation. This may be reflected in how safety skills and safety inoculation relate to the motivational impact of the intervention. Furthermore, earlier studies (Hedlund et al., 2016; Rodrigues et al., 2018; Burke et al., 2006; Williams et al., 2010) indicate that active participation on the part of the learners is important in terms of the impact of safety training on motivational, attitudinal, and safety knowledge outcomes. Therefore, utilizing active learning methods in safety training programs may play an important role in achieving positive, intervention outcomes. Finally, earlier studies indicate that a supportive learning environment plays an important role in student motivation, engagement and learning (see Shernoff et al., 2017). Bandura (1997) also stresses the role of emotional arousal in predicting self-efficacy beliefs. Thus, it is expected that fostering a positive atmosphere contributes to intervention impacts.

Based on the earlier studies presented above, it can be expected that implementing the *Attitude to Work* core components with fidelity contributes to positive, intervention outcomes. However, a more detailed analysis of the relationships between the different components and the different outcome variables is needed (Abry et al., 2017). It is possible that a single core component may play a central role for one outcome variable while its role may be less important for others. The goal of this study was to acquire knowledge for future refinement, adaptation and scale-out of the *Attitude to Work* program to help prevent injuries and illnesses among younger workers.

### Method

**Fig. 1:**
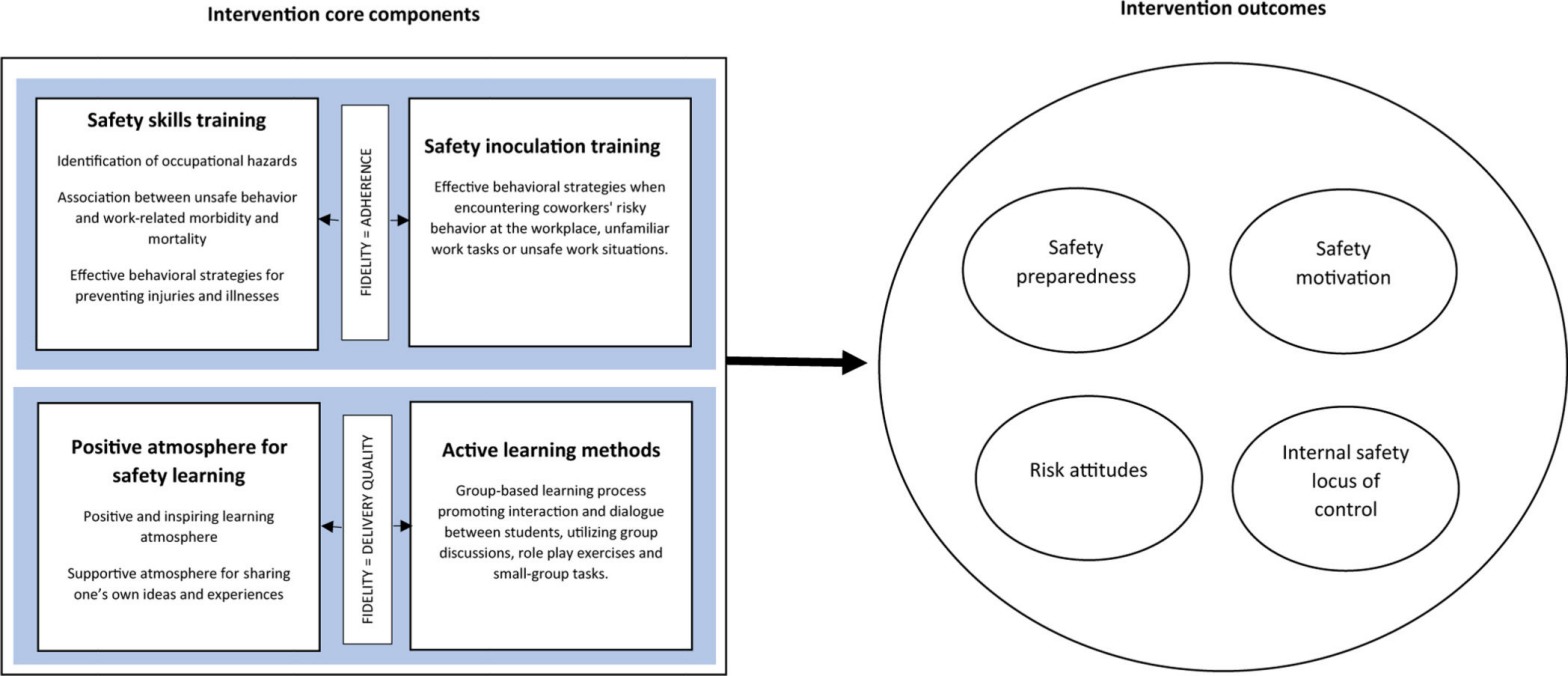
A representation of intervention core components (on the left side of the figure) associated with measured outcomes (right). Fidelity of implementation (on the left) is defined as adherence to the educational content and quality of intervention delivery. When delivered with fidelity, the intervention core components illustrated in the figure are hypothesized to be the main contributors to the outcomes of interest

This study uses data collected during a school-based cluster randomized controlled trial (RCT), conducted in 2015 in eight Finnish upper secondary-level vocational schools. Information about how the trial was conducted and the efficacy of the *Attitude to Work* program has been reported in previously published studies (Nykänen et al., 2018, 2019). In the current study, we explored the associations between the core components and student outcome variables (safety preparedness, internal safety locus of control, risk attitudes, and safety motivation) to identify the active ingredients of the intervention. Figure 1 illustrates the associations between the concepts explored in the study.

### Study Participants

In Finland, upper secondary vocational education concerns learning practical and work-specific skills in various occupational fields. The upper secondary vocational qualification takes approximately 3 years to complete. Students with this qualification are considered to have the basic vocational competence required for working life. Overall, 464 students from eight vocational schools participated in the previous RCT (Nykänen et al., 2018). The students were clustered in 44 student groups, the sizes of which ranged from 6 to 22 in the intervention condition (*M* = 13.6, *SD* = 5.2) and from 6 to 26 in the control condition (*M* = 12.7, *SD* = 5.0). The students in the control group did not participate in the program and did not provide study data for the analyses regarding association between intervention core components and targeted outcomes. Thus, the analyses presented here include only intervention condition participants (*n* = 229 students clustered in 22 groups, each with one teacher).

### Measures

#### Implementation Fidelity

In our study, we focused on student perceptions of the implementation fidelity of the hypothesized core components of the *Attitude to Work* program. Fidelity of delivery of prespecified intervention active ingredients was evaluated using a revised version of a measurement technique developed by Vuori et al. (2005). This approach is consistent with previous school-based prevention studies (Abry et al., 2017; Bast et al., 2019; Dusenbury et al., 2003; Guerin et al., 2019). Our study evaluated implementation fidelity, assessed as two measures of adherence to the program (related to the acquisition of safety skills training and safety inoculation training) and two measures of quality of teacher delivery (related to positive learning atmosphere and the active learning facilitated by the teacher) to the four intervention core components at follow-up. The students rated various aspects of the implementation of the intervention process using a five-point scale, ranging from (1) “not at all” to (5) “A great deal.” The safety skills training dimension included five items with *α* = 0.86 (How much did you practice how to... Example item: *Seek information and support at the workplace?*). Safety inoculation training included three items with *α* = 0.84 (How much did you discuss solutions for the following situations? Example item: *Employee is not sure how to perform the work task*). The positive learning atmosphere included three items with α = 0.84 (To what extent...Example item: *was the atmosphere positive and inspiring during the training*), and the active learning dimension was measured using five items with *α* = 0.78 (How much did...Example item: *you work in small groups*). A detailed description of the questionnaire items is presented in the Online Resource A.

#### Safety Preparedness

The safety preparedness measure is a nine-item measure of safety self-efficacy and safety inoculation. Six, 5-point (1 = very poorly; 5 = very well) self-efficacy items addressed safety-related activities at the workplace (example item: “acquiring instructions or guidelines at work in order to work safely”). Three, 5-point inoculation items (1 = very few; 5 = many) measured the extent to which participants had ideas or plans for situations in which they may encounter various safety-related problems in the workplace (example item: “Co-workers’ attitudes and behavior are harmful to occupational safety”). A more detailed description of the safety preparedness scale is provided in Nykänen et al. (2018). The internal consistency reliability (*α*) at baseline was 0.84 and at follow-up was 0.87.

#### Internal Safety Locus of Control

Internal safety locus of control was measured using a three, 5-point (1 = strongly disagree; 5 = strongly agree) items (example item: “People can avoid injury if they are careful and aware of potential dangers”) adapted from a measure by Mazaheri et al. (2012), *α* = 0.67 at time 1 and *α* = 0.63 at time 2.

#### Safety Motivation

In our study we used a 5-point (1 = strongly disagree; 5 = strongly agree) scale developed previously by Neal et al. (2000), consisted of three items (example item: “I feel that it is important to maintain safety at all times”), *α* = 0.86 at baseline and at follow-up *α* = 0.84.

#### Risk-Taking Attitude

The measure used in the current study measure was adapted from the general safety attitude scale developed by Henning et al. (2009). The five-point scale (1 = strongly disagree; 5 = strongly agree) included three items (example item: “Sometimes it is necessary to take risks to get a job done”) that measured the extent to which participants viewed occupational safety-related risk-taking as appropriate at the workplace. At both baseline and follow-up, *α* = 0.81 for the risk attitude measure.

#### Procedure

Student participants (229 students in 22 groups) participated in the *Attitude to Work* intervention program implemented during the 2015 school year. Each school received a two-day teacher training workshop and the intervention program was implemented at school by the teachers within approximately 2 weeks of student baseline measurements. The intervention program consisted of 2 days of training and lasted 12 h in total. The students completed the follow-up questionnaires immediately after completing the *Attitude to Work* program.

#### Analyses

We explored the correlations, means, and standard deviations of the study variables using individual-level data. To describe the magnitude of intervention effects, we calculated effect sizes for between-group (intervention vs. control condition) differences regarding mean pre-post changes using the *d*_ppc2_ formula by Morris (2008) which represent the standardized mean difference between intervention and control group. Effect size calculation is based on earlier intervention efficacy evaluations (Nykänen et al., 2018, 2019). Group-level variables were the focus of the analyses. Previous studies have described that student ratings can be aggregated at the student-group level to yield a measure of the “shared perception of the environment” (Lüdtke et al., 2009). Aggregated student group average variables refer to the shared perceptions of the group as a whole. In our study, we aggregated student-level responses as to their perceptions of teachers’ implementation of intervention core component variables to form collective, group-level variables. Before aggregating these data, we calculated intra cluster correlations (ICC1 and ICC2) and within-group agreement statistics (RWG_j_) for all the group-level variables to justify the use of aggregated variables in the models.

We conducted confirmatory factor analysis using Mplus 7.4 software (Muthén and Muthén, Los Angeles, CA) to examine the proposed four-factor scale. We assessed model fit using the chi-square index (*χ*_2_), the Tucker-Lewis index (TLI), the standardized root-mean-square residual (SRMR), the comparative fit index (CFI), and the root mean square error of approximation (RMSEA). Good model fit was evaluated using the following benchmarks: root-mean-square error of approximation (RMSEA ≤ 0.06), standard root-mean-square residual (SRMR < 0.08), and comparative fit index (CFI ≥ 0.95; Hu & Bentler, 1999). To test the hypothesis that the intervention’s core components had positive associations with the intervention’s outcomes, we estimated 16 separate models using generalized linear mixed models (GLMM) in SPSS version 25. The potential effect of clustering due to student groups was taken into account by using a random intercept model. Each model included a different core component and intervention outcome. All models were adjusted for baseline outcome values.

## Results

### Descriptive Statistics

Table 1 shows the means and the correlations of the study variables using an individual level of analysis. Standardized effect sizes were 0.33 for safety preparedness, 0.22 for internal safety locus of control, 0.17 for safety motivation, and 0.22 for risk attitudes indicating small intervention effects. The group-level properties regarding ICC(1) and ICC(2) of the intervention’s core components across the intervention condition student groups (*n* = 22) are presented in Table 1. The mean RWg_(j)_ values were as follows: for safety skills training, 0.79 (ranged between 0.67 and 0.92); for safety inoculation training 0.76 (ranged between 0.59 and 0.88); for positive learning atmosphere 0.74 (ranged between 0.46 and 0.92); and for active learning techniques 0.72 (ranged between 0.49 and 0.91). Thus, mean RWg_(j)_ values (> 0.70) indicated generally accepted agreement levels in core component measures.

### Association Between Core Components and Intervention Outcomes

Confirmatory factor analysis regarding intervention fidelity measurement indicated an acceptable four-factor model fit (*χ*^2^[98] = 202.294, *p* < 0.001, CFI = 0.940, TLI = 0.926, SRMR = 0.049, RMSEA = 0.067 [95% confidence interval = 0.054–0.080]). All the factor loadings of the manifest indicators were significant (*p* < 0.001) and were between 0.46 and 0.87. The standardized factor loadings of the intervention fidelity measurement are presented in the Online Resource A.

Table 2 presents the results of 16 multilevel models exploring the association between core components and intervention outcomes. The results of our analyses demonstrated positive associations between the intervention’s core components and the student outcomes. Safety skills training had statistically significant associations with safety preparedness (*b* = 0.52, *p* < 0.01), internal safety locus of control (*b* = 0.65, *p* < 0.01), and safety motivation (*b* = 0.37, *p* < 0.05). Safety inoculation training had similarly statistically significant associations with safety preparedness (*b* = 0.28, *p* < 0.05) and internal safety locus of control (*b* = 0.30, *p* < 0.05) but not with safety motivation. We also found that a positive learning atmosphere had a statistically significant relationship with safety preparedness (*b* = 0.17, *p* < 0.05) and safety motivation (*b* = 0.20, *p* < 0.05). Finally, active learning techniques had a positive association only with safety motivation (*b* = 0.18, *p* < 0.05). Contrary to our expectations, we found no statistically significant relationship between any of the core components and risk-taking attitudes.

## Discussion

Given that schools have limited resources for implementing new prevention programs, ready to-use, evidence-based programs are important for increasing the adoption and implementation of OSH training and education in school-based contexts (Guerin et al., 2019). Our study highlights that the focus on adherence to the educational content is not enough, but attention should also be paid to the manner which the evidence-based safety training programs in schools are implemented. Overall, our results demonstrated that the fidelity measurement of adherence related to the acquisition of safety skills training was the strongest active ingredient in *Attitude to Work* safety training program. Adherence related to safety inoculation training had similar associations regarding competence-related beliefs, but the results showed no significant relationship with safety motivation. This result may be related to the limited work experience of young people. A lack of previous personal experiences on safety related barriers may affect an individual’s perception of the importance of advance preparation for them. The positive connection of safety skills training and safety inoculation training to competence-related beliefs can be interpreted through the lens of social-cognitive theory. Students identify and practice behavioral strategies to prevent work-related morbidity and mortality and to overcome barriers for safe work. During these training activities, students are given the opportunity to notice the development of their safety skills (mastery experiences) and can observe peers as they engage in learning process (vicarious learning). Students are also guided toward identifying controllable causes of work-related incidents which in turn can have a positive impact on perceived control over safety.

The study results suggest that fostering a positive and supportive learning atmosphere contributes to motivational impact of safety training activities. Moreover, we found that utilizing active learning techniques were related to safety motivation. This result is comparable with a study by Hedlund et al. (2016) that demonstrated that interventions where the participants had a high degree of participation led to increased safety motivation. In our study, quality of delivery in terms of utilizing active learning methods demonstrated no associations with other outcome variables. These results can be interpreted as that the benefits of active learning methods in school-based safety training may relate to motivational outcomes, but for competence-related beliefs, such as internal safety locus of control and safety preparedness, the delivery of educational content with high fidelity perhaps may play a more central role.

A previous RCT (Nykänen et al., 2018) indicated that the risk attitudes of students who participated in the *Attitude to Work* training program decreased more than those of the students in the control condition. However, we found no statically significant associations between intervention core components and risk attitudes. This suggests that the intervention effect on attitudes was influenced by an unmeasured factor not considered in the prespecified intervention model (see Rojas-Andrade & Bahamondes, 2019). According to Damschroder et al., (2009), intervention implementation is “a social process that is intertwined with the context in which it takes place.” The safety training program is based on a dialogue between students and therefore may have activated a process of constructing more positive social norms among student participants. The injunctive safety norm concept provides one perspective on this matter. Injunctive safety norms refer to the extent to which individuals perceive others’ approval and expectations of safety-related behavior (Fugas et al., 2011). Previous studies have demonstrated that young workers’ risk-taking orientation is influenced by peer workers behaviors (Westaby & Lowe, 2005). Furthermore, Reid and Aiken (2013) found that changes in injunctive norms and normative feedback had a positive impact on attitude toward sun protective behaviors. Research from Pek et al. (2017) also provides empirical support for how injunctive safety norms may be associated with young workers’ job-related risk-taking behaviors. Hence, it possible that the group-learning process and safety related communication between students had an impact on perceived injunctive safety norms which in turn was the key factor in facilitating attitudinal changes. However, our fidelity measures did not assess the perceptions of injunctive safety norms. Further research is needed to explore this topic, including by measuring injunctive safety norms before and after safety training based on the peer learning process.

### Study Limitations

One study limitation is that we had no observational data on the implementation process or teacher reports on the delivery of the program core components. Also, our analyses indicate low group mean reliability for safety skills training and safety inoculation training measures. The relatively small student group sizes may have had an impact on the ICC values in our study data (Bliese, 2000). Finally, standardized effect sizes indicate only small intervention effects. However, even small intervention effects can be practically important if the intervention program is cost efficient and scalable (see Bakker et al., 2019). School-based interventions have the potential to reach a large number of young people entering working life and *Attitude to Work* program offers a ready-to-use, free, and publicly available training method for preparing future workers for safe and healthy employment.

### Future Research in Prevention Science

The current study advances prevention science by identifying the active ingredients of the evidence-based, *Attitude to Work* intervention, implemented in Finnish vocational school settings, that helps protect young workers from work-related morbidity and mortality.

Future research should explore interactions between the intervention active ingredients. In terms of the *Attitude to Work* training program, a positive learning atmosphere may be more supportive of active learning during small group assignments and role-play exercises. Furthermore, to better understand the mechanisms of change school-based interventions, it is important to consider the interplay between the intervention process and contextual factors. The delivery, with fidelity, of intervention core components may interact with the social context and produce unplanned intervention effects. This perspective is in line with earlier research (Kelly, 2012) that found that implementation evaluation should increase understanding of relevant contextual processes that play important role in intervention effectiveness. Exploring the interaction between the intervention program and the context may require a more holistic approach, such as by utilizing a mixed-methods design, to obtain a fuller picture of implementation processes. Finally, a discussion of balancing fidelity and adaptation to meet the local needs and constraints of program providers and recipients (Allen et al., 2018; Stirman et al., 2019) is a topic of scholarly interest in prevention science (see e.g., Bopp et al., 2013; Castro et al., 2004). Research should be focused on how the *Attitude to Work* intervention may be modified to fit different school, cultural, and other contexts and stakeholder needs to ensure that the future workforce is equipped with the knowledge, skills, and abilities they need to stay safe and healthy on the job.

## Supplementary Material

Suppl Material

## Figures and Tables

**Table 1:** Means, group-level properties, standard deviations and correlation coefficients (Pearson’s *r*) of study variables

Variables	M	SD	ICC(l)	ICC(2)	1	2	3	4	5	6	7	8	9	10	11	12

1 Safety preparedness T1	3.55	0.50	-	-	1											
2 Safety preparedness T2	3.68	0.54	-	-	0.65**	1										
3 Internal safety locus of control T1	4.12	0.54	-	-	0.27**	0.25**	1									
4 Internal safety locus of control T2	4.19	0.53	-	-	0.14*	0.38**	0.38**	1								
5 Safety motivation T1	4.31	0.65	-	-	0.28**	0.29**	0.48**	0.29**	1							
6 Safety motivation T2	4.51	0.53	-	-	0.34**	0.54**	0.30**	0.47**	0.42**	1						
7 Risk attitudes T1	2.53	0.92	-	-	−0.08	−0.20**	−0.28**	−0.22**	−0.33**	−0.37**	1					
8 Risk attitudes T2	2.22	0.90	-	-	−0.14*	−0.36**	−0.29**	−0.29**	−0.35**	−0.49**	0.65**	1				
9 Safety skills training	4.43	0.52	0.05	0.44	0.22**	0.40**	0.21**	0.35**	0.31**	0.36**	−0.24**	−0.22**	1			
10 Safety inoculation training	4.18	0.65	0.09	0.56	0.35**	0.45**	0.21**	0.34**	0.26**	0.40**	−0.08	−0.11	0.59**	1		
11 Supportive atmosphere	4.26	0.71	0.24	0.80	0.26**	0.43**	0.26**	0.35**	0.34**	0.42**	−0.16*	−0.23**	0.44**	0.48**	1	
12 Active learning methods	4.14	0.64	0.33	0.86	0.29**	0.41**	0.22**	0.28**	0.30**	0.39**	−0.17*	−0.21**	0.45**	0.50**	0.77**	1

**Table 2:** Summary of 16 multilevel models exploring the association between intervention core components and student outcomes

	Safety preparedness		Internal safety locus of control		Risk-taking attitudes		Safety motivation	
			
	Estimate	95% CI	Estimate	95% CI	Estimate	95% CI	Estimate	95% CI

Group-level safety skills training	0.52**	0.27, 0.77	0.65**	0.34, 0.97	−0.26	−0.82, 0.29	0.37*	0.06, 0.69
Group-level safety inoculation training	0.28*	0.06, 0.50	0.30*	0.03, 0.57	0.02	−0.39, 0.44	0.18	−0.07, 0.44
Group-level positive learning atmosphere	0.17*	0.02, 0.31	0.14	−0.04, 0.33	−0.11	−0.39, 0.15	0.20*	0.03, 0.36
Group-level active learning techniques	0.13	−0.01, 0.28	0.11	−0.07, 0.30	−0.17	−0.44, 0.08	0.18*	0.03, 0.34

Student group was included as a random effect to account for clustering in all study models. All models were adjusted for baseline outcome value
