# Supplementary material for: Identifying the “Active Ingredients” of a School-Based, Workplace Safety and Health Training Intervention
Source: Prev Sci. Author manuscript; Available in PMC 2022 Oct 1. (PMC8458184; doi:10.1007/s11121-021-01209-8)
Supplement: Suppl Material [file NIHMS1680327-supplement-Suppl_Material.pdf]

**Identifying the "active ingredients" of a school-based, workplace safety and health training intervention**

Mikko Nykänen, Rebecca J. Guerin & Jukka Vuori

**Online resource A**. Standardized factor loadings of intervention core components

| Core component | Core component evaluation questionnaire items | Standardized factor loading |
|---|---|---|
| Safety skills training *(How much did you practice…?)* | how to identify occupational hazards. | 0.788 |
| | how to prevent occupational hazards or incidents. | 0.841 |
| | safe working methods | 0.686 |
| | how to report dangerous situations or safety deviations | 0.825 |
| | how to seek information and support at the workplace | 0.691 |
| Safety inoculation training *(How much did you discuss solutions for the following situations…?)* | unexpected event or machine malfunction at the workplace | 0.849 |
| | negative attitudes towards occupational safety at the workplace | 0.878 |
| | employee is not sure how to perform the work task | 0.695 |
| Positive learning atmosphere *(To what extent…?)* | was the atmosphere positive and inspiring during the training | 0.784 |
| | were you inspired to improve occupational safety | 0.838 |
| | was it easy for to talk about your own ideas or experiences | 0.762 |
| Active learning techniques *(How much did …?)* | you work in small groups | 0.644 |
| | you did role-playing exercises that simulated practical situations | 0.464 |
| | you get involved in planning ways to improve occupational safety | 0.740 |
| | the trainer ask for your ideas or suggestions during the exercises | 0.801 |
| | the trainer ask you questions that encouraged discussion | 0.840 |
